# The Molecular and Cellular Effect of Homocysteine Metabolism Imbalance on Human Health

**DOI:** 10.3390/ijms17101733

**Published:** 2016-10-20

**Authors:** Henrieta Škovierová, Eva Vidomanová, Silvia Mahmood, Janka Sopková, Anna Drgová, Tatiana Červeňová, Erika Halašová, Ján Lehotský

**Affiliations:** 1 Biomedical Center Martin, Department of Molecular Medicine, Jessenius Faculty of Medicine in Martin, Comenius University in Bratislava, 03601 Martin, Slovakia; henrieta.skovierova@gmail.com (H.Š.); silvia.mahmood@jfmed.uniba.sk (S.M.); jankasopkovamt@gmail.com (J.S.); halasova@jfmed.uniba.sk (E.H.); 2 Department of Medical Biochemistry, Jessenius Faculty of Medicine in Martin, Comenius University in Bratislava, 03601 Martin, Slovakia; drgova@jfmed.uniba.sk (A.D.); lehotsky@jfmed.uniba.sk (J.L.); 3 Biomedical Center Martin, Department of Neurosciences, Jessenius Faculty of Medicine in Martin, Comenius University in Bratislava, 03601 Martin, Slovakia; 4 Department of Public Health, Jessenius Faculty of Medicine in Martin, Comenius University in Bratislava, 03601 Martin, Slovakia; mt.cervenova@uvzsl.sk; 5 Department of Medical Biology, Jessenius Faculty of Medicine in Martin, Comenius University in Bratislava, 03601 Martin, Slovakia

**Keywords:** homocysteine metabolism, hyperhomocysteimenia, cellular toxicity, oxidative stress, disease

## Abstract

Homocysteine (Hcy) is a sulfur-containing non-proteinogenic amino acid derived in methionine metabolism. The increased level of Hcy in plasma, hyperhomocysteinemia, is considered to be an independent risk factor for cardio and cerebrovascular diseases. However, it is still not clear if Hcy is a marker or a causative agent of diseases. More and more research data suggest that Hcy is an important indicator for overall health status. This review represents the current understanding of molecular mechanism of Hcy metabolism and its link to hyperhomocysteinemia-related pathologies in humans. The aberrant Hcy metabolism could lead to the redox imbalance and oxidative stress resulting in elevated protein, nucleic acid and carbohydrate oxidation and lipoperoxidation, products known to be involved in cytotoxicity. Additionally, we examine the role of Hcy in thiolation of proteins, which results in their molecular and functional modifications. We also highlight the relationship between the imbalance in Hcy metabolism and pathogenesis of diseases, such as cardiovascular diseases, neurological and psychiatric disorders, chronic kidney disease, bone tissue damages, gastrointestinal disorders, cancer, and congenital defects.

## 1. Homocysteine Metabolism

**Figure 1:**
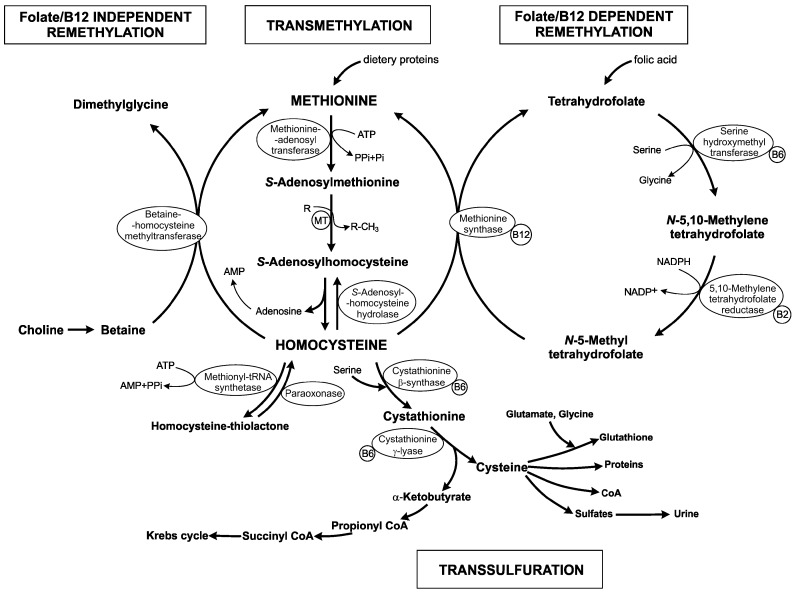
The schematic overview of homocysteine metabolism and its relationship with folic acid and vitamins. ATP: adenosine triphosphate; AMP: adenosine monophosphate; PPi: pyrophosphate; Pi: orthophosphate; B2/B6/B12: vitamins B2/B6/B12; CoA: coenzyme A; R: acceptor; R-CH_3_: methylated product; MT: methyltransferases.

Homocysteine (Hcy) is a non-essential, sulfur-containing, non-proteinogenic amino acid. It is synthetized by transmethylation of the essential, diet-derived amino acid methionine (Met) (Figure 1). It represents the only way through which Hcy is produced in humans. This conversion involves three steps, catalyzed by the following enzymes: *S*-adenosyl-l-methionine (SAM) synthetase/l-methionine adenosyltransferase (EC 2.5.1.6), methyltransferase (MT) and *S*-adenosyl-l-homocysteine (SAH) hydrolase (EC 3.3.1.1) that occur in different tissues. SAM synthetase activates Met in a reaction with ATP leading to SAM synthesis. SAM is used as a universal methyl donor not only in a variety of cellular biosynthesis of different compounds (i.e., creatine, epinephrine, carnitine, phospholipids, proteins, nucleic acids and polyamines) but also in epigenetic modulations, as regulation of DNA methylation (nuclear and mitochondrial), chromatin remodeling, RNA editing, noncoding RNA, micro RNA and post-translational modification of histones. SAH is the product of all SAM-dependent transmethylation reactions. It is well known that hypermethylation of several genes, based on higher SAM level, is likely linked with cancer [1,2].

There are several methyltransferases in mammalian cells, but phosphatidylethanolamine *N*-methyltransferase (PEMT; EC 2.1.1.17) in phosphatidylcholine synthesis and guanidine-acetate *N*-methyltransferase (GAMT; EC 2.1.1.2) for creatine synthesis are major contributors to the production of Hcy, mostly in liver. It is widely accepted that increased level of Hcy in plasma is linked with cardiovascular diseases or homocystinuria. Approximately 85% of all SAM-dependent transmethylations are due to these two MTs [3]. Hepatic PEMT serves as an additional route to generate phosphatidylcholine from phosphatidylethanolamine, instead of its direct synthesis from choline. The biosynthetic pathway, which is catalyzed by PEMT, requires three methyl groups from SAM. It was explored that PEMT represents the main consumer of SAM-derived methyl groups and production of Hcy [4,5]. Another important enzyme that transfers SAM-derived methyl group and subsequently results in Hcy production is glycine *N*-methyltransferase (GNMT; EC 2.1.1.20). GNMT is a cytosolic protein that is responsible for glycine methylation and generation of sarcosine. This enzyme has also a regulatory role. In liver, GNMT regulates the ratio of SAM/SAH as a means to optimize transmethylation reactions [6].

Hcy is a branching point of three major pathways, located mostly in the liver: (i) resynthesis to SAH through reversal activity of SAH hydrolase; (ii) remethylation to methionine by folate/B12-dependent/independent pathways; and (iii) transsulfuration to cystathionine (Figure 1).

After SAM-dependent transmethylation, SAH is rapidly metabolized by SAH hydrolase to adenosine and Hcy, which potentially increases Hcy concentration. Another consequence of an elevated Hcy level in cells is the accumulation of SAH in reaction of the reverse catalysis by SAH hydrolase. The increased concentration of SAH has strong negative effect on most of MT in mammal because SAH behaves as a potent allosteric inhibitor of these enzymes [7]. The intracellular ratio of SAM/SAH determines as an index of transmethylation potential [8]. Dysregulation in methylation status (i.e., hypomethylation) due to reduced synthesis of SAM is among the central mechanisms that explain the negative effect of hyperhomocysteinemia related to vascular diseases and neurodegenerative disorders where synthesis in neurotransmitters and proteins important for the structural integrity of brain is disrupted [9].

Under normal conditions, approximately 50% of Hcy is remethylated to form Met. Two distinct routes exist for the remethylation of Hcy back to Met to complete the methyl cycle. The first reaction is dependent on the presence of B vitamins and folate. The folate in the form as a coenzyme *N*-5-methyl tetrahydrofolate (THF) can donate a methyl group to Hcy in a reaction catalyzed by the vitamin B12-dependent enzyme methionine synthase (MS; EC 2.1.1.13). Thus, both folate and vitamin B12 status play an important role in Hcy balance within the cell and subsequently the plasma level circulation. It should also be noted that sufficient supply of *N*-5-methyl THF for folate-dependent remethylation of Hcy is part of “one-carbon” metabolism [10]. *N*-5,10-methylene THF reductase (MTHFR; EC 1.5.1.20) catalyzes the synthesis of *N*-5-methyl THF from *N*-5,10-methylene THF. This reaction requires NADPH, being regulated by SAM and SAH as a negative and positive regulator, respectively.

The second route for Hcy remethylation is independent of the “one-carbon” metabolism. It uses betaine as a methyl group donor, which is synthetized from choline by betaine-homocysteine *S*-methyltransferase (BHMT; EC 2.1.1.5). BHMT-dependent remethylation of Hcy occurs primarily in liver, kidney and lens, whereas the folate/vitamin B12-dependent route is found universally in all tissues. For folate-independent methionine remethylation, the regulation and/or expression of BHMT has been shown to affect Hcy concentrations in plasma with the wide clinical manifestations.

The last disposal pathway of Hcy is its transsulfuration to cysteine (Cys). The first reaction is a condensation between Hcy and serine (Ser) leading to cystathionine production, which is further hydrolyzed to Cys and α-ketobutyrate. These two reactions are catalyzed by the B6-dependent enzymes cystathionine β-synthase (CBS; EC 4.2.1.22) and cystathionine γ-lyase (CSE; EC 4.4.1.1), respectively. α-Ketobutyrate is proceed during oxidative decarboxylation to propionyl~CoA, which is converted to succinyl~CoA, one of the intermediate of the Krebs cycle. The transsulfuration pathway is responsible for both: (i) Met catabolism; and (ii) sulfur atom transfer from Met to Ser, yielding Cys. Cys is a precursor for the synthesis of proteins, coenzyme A, sulfates and glutathione. The last one is a tripeptide that reduces reactive oxygen species, thereby protecting cells from oxidative stress. The other excessively important enzymatic activity of transsulfuration enzymes (CBS, CSE) is H_2_S production from catalysis of Hcy and/or Cys. H_2_S is the third gasotransmitter and is produced in different cells and tissues in human body. It alone or with two other gasotransmitters (NO and CO) regulates an array of physiological processes [11]. The impairment of transsulfuration processes is associated with homocystinuria, autism, cirrhosis, immune dysfunction or pancreatitis [12,13,14,15].

Hcy is formed in all tissues. However, its detoxification through the transsulfuration pathway occurs in liver, kidney, small intestine, pancreas and lens. The brain and adipose tissue contain CBS but lack CSE. Furthermore, the central nervous system lacks BHMT, being thus dependent on folate/vitamin B12-dependent pathway for the conversion of Hcy to Met, which refers brain to higher vulnerability to the increased Hcy levels [16]. In this condition, the central nervous system is attacked by both cerebrovascular alterations and brain parenchyma disorders.

## 2. Hyperhomocysteinemia, an Elevated Level of Homocysteine in Plasma

**Figure 2:**
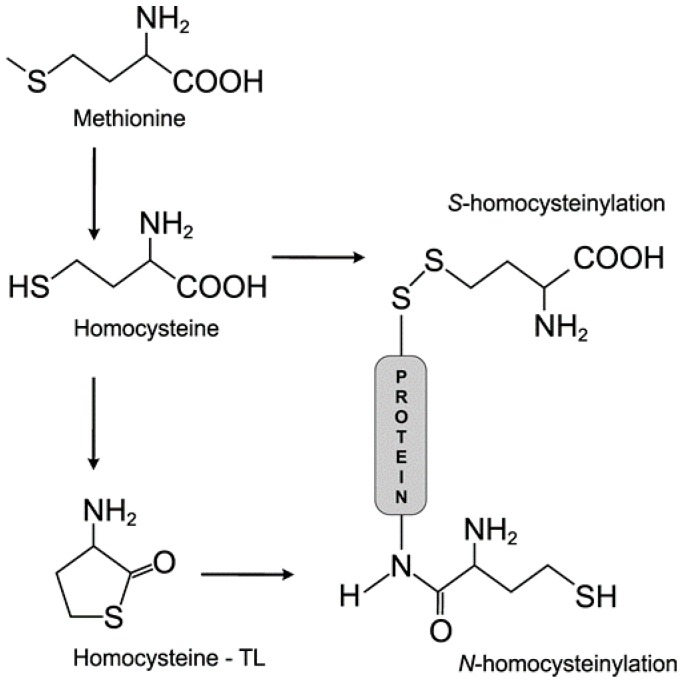
The structure of methionine, homocysteine, homocysteine-thiolactone (homocysteine-TL; Hcy-TL) and *N*-/*S*-homocysteinylation of proteins. Methionine is metabolized to homocysteine which should be subsequently catalyzed to Hcy-TL by methionyl t-RNA synthetase. Hcy-TL modifies proteins by *N*-homocyteinylation. On the other hand, Hcy could bind to cysteine residues of a protein to make disulfide bound resulted in *S*-homocysteinylation of proteins.

There are several forms of plasma Hcy: (i) free Hcy; (ii) protein-bound Hcy (*S*-linked, and *N*-linked); (iii) oxidized forms of Hcy; and (iv) Hcy-thiolactone (Figure 2) [17]. Under physiological conditions, less than 1% of total Hcy (tHcy) is present in a free reduced form (SH group) in plasma. About 10%–20% of tHcy has been found in different oxidized forms, i.e., Hcy-Cys and homocysteine (the Hcy dimer) in plasma. However, the majority of plasma tHcy (80%–90%) is *N*-linked and *S*-linked to γ-globulins or serum albumin [18]. Plasma tHcy is defined as the pool of free Hcy, protein-bound Hcy, homocystine and as well as Hcy bound to Cys by disulfidic bond. tHcy is used as a predictive risk factor for cardiovascular disorders, the stroke progression, screening for inborn errors of Met metabolism, and as a supplementary test for vitamin B12 deficiency. The methods used to determine Hcy level in different biological samples can be classified into chromatographic methods, enzymatic assays and combined assays (enzymatic reaction followed by an immunoassay). The concentration of tHcy can also be assessed through capillary electrophoresis with a suitable detection system. Different methods used to assess tHcy and Hcy-thiolactone concentration in biological samples (plasma, urine) are very nicely summarized by Manolescu et al. [19].

The reference of tHcy in plasma is in range 5–10 μM in human. Under normal conditions, plasma Hcy concentrations do not exceed 15 μM [20]. Elevation of plasma Hcy is manifested as hyperhomocysteinemia (hHcy). Several types of hHcy are classified in relation to the tHcy concentration: moderate (16–30 μM), intermediate (31–100 μM), and severe (higher than 100 μM) [21]. Severe hHcy occurs in homocystinuria, an innate metabolic disorder characterized by a deficiency of CBS enzyme activity. Affected patients exhibit plasma concentrations of Hcy that can reach up to 500 μM. Generally, the accumulation of Hcy has been resulted from the inability to regulate its pathway and can be attributed to endogenous factors (polymorphisms of the genes coding enzymes involved in Hcy metabolism such as CBS, MS, and MTHFR) and/or exogenous factors (dietary deficiency of folate, vitamins B6 or B12 and also the intake of proteins rich in Met and Cys) [22]. These dietary nutrients influence the supply of methyl groups and regulate the biochemical pathways for methylation processes. Supplementation with natural folate-rich foods, folic acid and *N*-5-methyl THF reached a similar reduction in Hcy concentrations [23]. The efficacy of *N*-5-methyl THF has been compared with that of folic acid in several studies with different results. Fohr et al. [24] showed that the supplementation with folic acid was more effective than with *N*-5-methyl THF to decrease plasma tHcy in women, while Venn et al. [25] reported that a low-dose of *N*-5-methyl THF was at least as effective as folic acid in reducing tHcy concentrations in healthy subjects.

With significant reduction in MTHFR enzymatic activity, Hcy cannot be remethylated to Met and accumulates. In humans, more than twenty different polymorphisms in *MTHFR* gene have been described until now, but two are the most common ones, C677T and A1298C [26,27]. The role of the *MTHFR* C677T polymorphism has been studied in different laboratories and its role as a risk factor for cardiovascular disease and ischemic stroke was established. Increased Hcy levels are associated with several disorders that affect central nervous system (CNS), however, another molecular variant of the *MTHFR*, G1793A was found to be associated with a different tumorigenesis in men [28].

Moreover, Hcy level increases with age, at least in part due to the increasing deficiency of vitamin B12 observed in the elderly, as result mostly by its poor absorption from food, and also due to declining renal function. Older people have higher prevalence of protein oxidation and DNA lesions revealing that the aging tissues such as endothelium or brain suffer from accumulated oxidative damage during life [29]. Thus, Hcy may act together with the other cellular changes associated with aging in development of cellular dysfunction.

## 3. Toxicity of Homocysteine

Over the years, different hypothesis focused on Hcy toxicity have been developed. However, despite the efforts, none of them does clearly explain the Hcy biotoxicity. The three main pathways of Hcy biotoxicity have been discussed in the literature: (i) protein structure modifications known as homocysteinylation; (ii) oxidative stress induction; and (iii) excitotoxicity.

### 3.1. Homocysteine Induces Homocysteinylation

The Hcy toxicity is proposed as consequence of covalent binding of this compound to proteins followed by modifying their functions (Figure 2). The process is called homocysteinylation and is considered as a posttranslational modification of proteins. The degree of protein homocysteinylation is proportional to the increased level of plasma Hcy [30]. *S*-homocysteinylation is when Hcy binds through its free thiol group to another free thiol group derived from a Cys residue in a protein molecule and makes the disulfide bound. These changes have a strong influence on the thiol-dependent redox status of proteins. *N*-homocysteinylation is a result of the high reactivity of Hcy-thiolactone (Hcy-TL) which synthesis is catalyzed by methionyl-tRNA synthetase (EC 6.1.1.10) in the presence of ATP. *N*-homocysteinylation occurs when Hcy interacts by its amino group with the ε-amino group of a lysine residue in protein and alters or impairs the structure and function of modified protein. Experimental studies have shown that Hcy-TL contributes to Hcy pathobiology, which is caused by protein *N*-homocysteinylation [31]. In vivo, Hcy-TL targets and modifies blood albumin, hemoglobin, immunoglobulins, LDL, HDL, transferrin, antitrypsin, and fibrinogen [17]. In addition, Hcy-TL behaves as Na^+^/K^+^-ATP-ase (EC 3.6.3.9) inhibitor in the hippocampus, cortex and brain cells of rats. It changes the membrane potential with detrimental effect on neuronal cells [32]. Elevated plasma levels of Hcy-TL and proteins modified by *N*-homocysteinylation are direct consequences of either genetic defects in Hcy metabolism or a methionine-rich diet [33]. Commonly, *N*-homocysteinylation affects the function of proteins through introduction of new free thiol groups and inactivation of free amino groups results into affecting the overall redox potential of proteins followed by increased oxidative stress [34]. The pathological consequences of *N*-homocysteinylation could result in cytotoxicity, most possibly through endoplasmic reticulum stress, activation of unfolded protein response, enhanced protein degradation, enzymatic inactivation and even in amyloid formation [35]. Besides, it was proven that proteins modified by *N*-homocysteinylation could act as neoantigens, triggering activation of the inflammatory response which is a key component of atherogenesis, atherotrombosis and stroke etiology. Moreover, these neoantigens induce an autoimmune response and the concentration of autoantibodies is higher in some human pathologies (i.e., cerebrovascular disease, renal failure) comparing to normal individuals [36]. *N*-homocysteinylated proteins in the luminal face of vascular endothelial cells are recognized by specific antibodies and this neoantigen–autoantibody interaction leads to the activation of circulating macrophages, which become responsible for repeated vascular endothelium damage. Furthermore, Hcy-TL impairs the ability of the vascular endothelium to regenerate itself by direct inhibition of lysyl oxidase (Lox; EC 1.4.3.13), which is responsible for the correct cross-linking of collagen and elastin in the arterial wall [37]. This has a strong impact to the vascular stiffness.

Gurda et al. [38] analyzed the changes in gene expression profiles induced by Hcy and its products using microarray technology, real-time quantitative PCR and bioinformatic analysis. They identified 47, 113, and 30 different mRNA regulated by *N*-homocysteinylated protein, Hcy-TL, and Hcy, respectively. Each metabolite induced a unique group of gene expression profile. Top molecular pathways affected by Hcy-TL were chromatin organization, lipid-related processes and “one-carbon” metabolism [38]. Top pathways of *N*-homocysteinylated protein and Hcy were blood coagulation, sulfur amino acid metabolism and lipid metabolism. It was suggested that top disease related to all three inductors was atherosclerosis and coronary heart disease [38].

### 3.2. Homocysteine and Oxidative Stress

One of the first hypothesis suggested that hydrogen peroxide (H_2_O_2_) formed in redox reactions involving the thiol group of Hcy was responsible for the toxicity of this compound. The major drawback of this hypothesis was that Cys (acts as a regular amino acid) is not a risk factor for vascular diseases, despite its up to 30-fold higher concentration than Hcy [39]. Oxidative stress is defined as an imbalance between the production of reactive species and antioxidant defenses. It can result from increased production of reactive species and reduced levels of antioxidants. Different studies showed that redox reactions may be a key factors in the development of atherosclerosis, vascular hypertrophy and thrombosis in the animals with hHcy [40,41]. Oxidative stress is generated during oxidation of the free thiol group of Hcy which binds via a disulfide bound to plasma proteins, mostly albumin, to other low molecular plasma thiols or to a second Hcy molecule. Hcy increased production of reactive oxygen species. They may form hydroxyl radicals which can remove electrons from other molecules and could induce the subsequent oxidation of lipids, proteins, carbohydrates and nucleic acids which can lead to the endothelial dysfunction, or to the vessel wall damages, followed by platelet activation and thrombus formation [34,42]. Autooxidation of Hcy metabolites leads to the accumulation of strong oxidizing agent, H_2_O_2_. The necrotic death of neurons was induced after long term incubation of cells with Hcy metabolites [43]. Accumulation of the oxidized biomolecules modifies the biological functions of many cellular pathways. Several mechanisms have been proposed for Hcy induced oxidative stress: (i) Hcy autoxidation; (ii) inhibition of the enzymatic activity of antioxidants in cells; (iii) disruption of extracellular superoxide dismutase from endothelial surfaces; (iv) activation of NADPH oxidases; and (v) nitric oxide synthase (NOS)-dependent generation of superoxide anion [44]. Moreover, reactive oxygen species and oxidative stress lead to the formation of nitrotyrosine, an indicator of nitric oxide and superoxide radical reaction, resulting in the formation of strong oxidant peroxynitrite. Peroxynitrite leads to tyrosine nitration, which causes the alteration in protein function and induces cellular dysfunction [45].

### 3.3. Homocysteine as a Neurotoxin

The toxic effect of Hcy on brain tissue is influenced by the absence two of the major metabolic routes for Hcy elimination: betaine-mediated conversion Hcy to Met and transsulfuration Hcy to Cys. In addition, Hcy acts an agonist for both groups of glutamate receptors, metabotropic (groups I and III) and ionotropic (α-amino-3-hydroxy-5-methyl-4-isoxazolepropionate (AMPA)) receptors, as well as for *N*-methyl-d-aspartate receptor (NMDA) [43]. Overstimulation of these receptors results in increased level of cytoplasmic calcium, higher production of free radicals and activation of caspases leading to apoptosis [19,43,46]. Hcy mediated NMDA receptor induction of neuronal cells could lead to their death due to transient activation of extracellular signal-regulated kinases (ERKs), mitogen-activated protein kinase (MAPK) and p38 MAPK [47,48] that is different from downstream signaling pathways triggered by other NMDA receptor agonists. Not only neuronal cells are exposed to toxic effects of Hcy, but glial cells too [49]. The importance of astrocytes in the brain homeostasis, assisting in neurogenesis, determining in micro-architecture of the grey matter, and also in energy metabolism has been well documented [50].

Moreover, hHCy can often results in intracellular Ca^2+^ mobilization and endoplasmic reticulum stress followed with the subsequent development of apoptotic events, remodeling of extracellular matrix in brain parenchyma and endothelial dysfunction [34,44,46]. In humans, the increased level of Ca^2+^ damages mitochondria by collapsing the mitochondrial membrane potential and the production of ATP is suppressed. Furthermore, the consecutive leakage of cytochrome c from mitochondria as well as reactive oxygen species activate the caspase 3 pathway which leads to DNA fragmentation, a hallmark of apoptosis [51,52].

It was proved that Hcy itself is able to induce blood-brain barrier (BBB) disruption [53]. This disruption can be due to several different processes. First, Hcy induces an imbalance between the activity of the matrix metalloproteinase 9 (MMP-9) and the tissue inhibitor of metalloproteinase 4 (TIMP-4), in the way of increasing MMP-9 and decreasing TIMP-4 activity [54]. Subsequently, MMP-9 interacts with different components of the BBB and leads to disruption of this structure. Second, Hcy acts as an excitatory neurotransmitter for: (i) γ-aminobutyric acid (GABA) receptors A, which leads to increased vascular permeability [55]; and (ii) NMDA receptor [43]. The expression of NMDA receptor is not confident to neurons only. In addition, endothelial cells from cerebral tissue are able to express this type of receptor. Free radicals are responsible to induce the activity of NR1 subunit of the NMDA receptor which is resulted in the increased susceptibility of these cells to excitatory amino acids and finally, in BBB disruption [43,56]. Several pro-inflammatory agents (i.e., endotoxins, cytokines, or oxidative stress factors) have an up-regulating effect on MMP-9 activity in astrocytes in vitro [57]. Anyway, the accumulation of toxic free radicals should play a key role in BBB disruption via the increased activity of MMPs [58].

## 4. Hyperhomocysteinemia and Diseases

Nowadays, the relationship between imbalances in Hcy metabolism and numerous pathological conditions is still not well understood. Hcy association with vascular diseases has been recognized since 1962 when Carson and Neil [59] identified metabolic abnormalities which caused mental retardation in patients with elevated levels of Hcy in the urine. They revealed a new disorder of Met metabolism referred to as homocystinuria. In 1969, McCully published a study showing on the vascular pathologies in patients with homocystinuria. It was the first time that elevated Hcy level was linked to premature vascular disease [60]. By the 1990s, an explosion of studies examining this hypothesis has brought hHcy as a risk factor for cardiovascular diseases into a whole new light. Today, it is widely accepted that an elevated level of Hcy (more than 15 μM) is an independent risk factor for cardio and cerebrovascular diseases. Recent studies have demonstrated a strong correlation between elevated Hcy levels and neurological disorders, chronic kidney disease, osteoporosis, gastrointestinal disorders, cancer and congenital defects development [44,61,62,63,64].

### 4.1. Cardiovascular Diseases

Hcy has been recognized as an independent risk factor for cardiovascular diseases (CVD) [60,65]. It has been estimated that a 2.5 µM rise in plasma tHcy concentrations is associated with 10% increase in CVD risk [66]. Moreover, it was shown that increased plasma tHcy levels (above 20 μM) should be associated with nine-fold increase of myocardial infarction and also with subsequent stroke risk comparing to concentration below 9 μM [19]. A key issue that remains to be resolved is whether a condition of hHcy has a direct, causal impact on vascular diseases, or exists as a biomarker that reflects another changes in metabolism for the adverse effects on vascular function. hHcy and the frequency of myocardial infarction are positively correlated, even after adjustment for another risk factors of CVD [67]; thus, Hcy should emerge as the “cholesterol” of the 21st century. Meta-analysis of more than 80 studies on folate metabolism and CVD has shown an association with hHcy and cardiovascular events, including vessel damage, atherosclerotic and thrombosis [68]. In contrast, the level of tHcy in plasma is not related to total CVD risk factor and should or should not be associated to CVD-related mortality [69]. However, it should be mentioned that clinical trials focused on Hcy metabolism with vitamin B supplementation to decrease Hcy concentrations have not been as effective as anticipated [70]. As was shown, Hcy has a negative effect on endothelial cells, revealing novel mechanisms involved in hHcy and cardiovascular health. In endothelial cells of the coronary arteries, hHcy appeared to result in reduced tetrahydrobiopterin function, which is an important cofactor for nitric oxide-derived vasodilation [71]. However, the impact of hHcy on vascular oxidation was not so significant comparing with the impact of *N*-5-methyl THF, which seems to regulate the balance of nitric oxide in blood vessel [72]. This could suggest that increased tHcy level may not only be a cause of endothelial dysfunction, but it should also be used as a biomarker for another complications [64]. The impact of hHcy on endothelial cell health could participate in the development of hypertension, because circulating elevated Hcy level was associated with the increased arterial stiffness in prehypertensive patients [73]. Very recently, it has been reported that the ratio between SAM/SAH should be used as a biomarker and may provide a sensitive indicator for the clinical diagnosis of atherosclerosis [74]. Another scientific group investigate the effect of elevated Hcy level on fatty acid binding protein 4 (FABP4) [75]. Their results showed that FABP4 has a very important function in lipid metabolism disturbance after Hcy treatment and also that DNA methyltransferase 1 (DNMT1; EC 2.1.1.37) could be a potential therapeutic target in Hcy-related atherosclerosis [75].

### 4.2. Neurological and Psychiatric Disorders

The relationship between Hcy and neurological problems, such as depression, Parkinson’s and Alzheimer’s diseases is now widely recognized. The major depressive disorder is generally linked to impaired transmission or lower levels of neurotransmitters (dopamine, norepinephrine and serotonin). In a recent longitudinal study of more than 11,000 patients, elevated Hcy concentration was associated with a 26% increase in the likelihood of depressive symptoms [76]. Diet supplemented with vitamins B2, B6, B12 and folic acid has been shown to effectively decrease plasma Hcy levels and reduce depressive symptoms [77]. It was shown that 10% to 30% of patients with Parkinson’s disease have elevated plasma tHcy level. Parkinson’s disease is a neurodegenerative disorder with loss of motor control which is often resulted in tremors. They are caused by damage of dopaminergic neurons. Enzyme, catechol-*O*-methyltransferase (COMT; EC 2.1.1.6) catalyzes transfer of methyl group from SAM to catecholamine’s neurotransmitters, such as dopamine, epinephrine and norepinephrine and makes them inactive. Hcy production in these reactions could be one of the explanation of hHcy in Parkinson’s patient. Hcy can also be linked to the progression of Alzheimer’s disease. Increased Hcy concentration could lead to elevate γ-secretase activity, accumulation of amyloid β and could support hyperphosphorylation of tau protein in the brain [78,79]. The increased permeability of the BBB precedes the beginning of cerebral pathology connected with the progression of Alzheimer’s disease in patients with moderate hHcy [80]. Moreover, the rate of cognitive decline positively correlated with the increased level tHcy in patients with the moderate stage of Alzheimer’s disease and they have stronger behavioral disturbances which could be associated with major depressive disorder [81,82]. Very recently was shown, that Hcy and Hcy-TL enhance the interaction between fibrinogen and amyloid β, promote the formation of tighter fibrin clots and delay clot fibrinolysis [83]. It has been also reported that dysfunctional folate-methionine pathway enzymes, mostly *MTHFR* polymorphisms C677T and A1298C, may play an important role in the pathophysiology of autism (MIM 209850) [84,85]. Rai [86] in his meta-analysis has compared results from 13 case control studies focused on autism and *MTHFR* C677T polymorphism. His report strongly suggested a significant association of *MTHFR* C677T polymorphism with autism [86].

### 4.3. Chronic Kidney Disease

Patients with chronic kidney disease (CKD) have a noticeably increased risk of CVD. Based on the meta-analysis by Matsushita et al. [87], the adjusted cardiovascular risk increases when the estimated glomerular filtration rate (eGFR) goes below 75 mL/min. Half of these patients die of CVD before reaching the last stages and are the most interesting, considering their relative number. Therefore, CKD can be considered as a coronary heart disease equivalent [88]. Patients with severe hHcy occur in homocystinuria, an inherited metabolic disorder characterized by a deficiency of CBS enzyme activity [89]. Affected patients exhibit extremely elevated plasma Hcy level (up to 500 μM), which could be associated with higher cardiovascular risk. Generally, the folate supplementation is used in hHcy treatment. Interestingly, the folate studies have shown negative with respect to cardiovascular risk, but they can have a positive effect on stroke and cognitive dysfunction [87,90]. It needs to be mentioned that many patients can receive folate supplementation in order to replace folates lost during dialysis. It has been shown that folate supplementation does not reduce the intracellular concentration of Hcy. Moreover, higher level of folates may even disturb the physiological regulation of intracellular “one-carbon” metabolism [91]. Another reason of non-effective folate treatment in CKD patients could be the down-regulation in the folate receptor expression [92].

### 4.4. Bone Tissue Damages

Hcy can also affect the proper osteoclast activity. In vitro, the cultivation of bone marrow cells with Hcy enriched media showed that Hcy up-regulates the formation of osteoclasts and on the other hand, suppresses apoptosis in these cells due to higher production of reactive oxygen species. In patients with hHcy, the elevated activity of osteoclasts will lead to increase in bone resorption followed by higher risk of fractures and decrease in bone mineral density [64]. Furthermore, elevated level of Hcy activates caspase-dependent apoptosis in human bone marrow stromal cells, resulted in impairing of bone repair [93]. It was found that hHcy in rats led to an increased accumulation of Hcy in bone tissue co-localized mostly in collagen extracellular matrix (65%). This accumulation of Hcy was associated with a “spongy” bone phenotype and corresponding to the decrease in bone strength [94]. Moreover, Hcy is a product of methylation reactions, which are included in epigenetic modifications. Thaler et al. [95] studied the expression of *Lox* gene, which encodes lysyl oxidase, an extracellular enzyme essential for collagen cross-linking and stability. They found that Hcy has a down-regulating effect on the *Lox* expression. This could indicate a novel mechanism for bone tissue damage resulting from hHcy.

### 4.5. Gastrointestinal Disorders

Increased plasma Hcy level has been implicated in a variety of gastrointestinal diseases, including constipation, Crohn’s disease, inflammatory bowel disease, and colorectal cancer [61,96]. hHcy is associated with inflammatory remodeling of gastrointestinal tract which could lead to increased production of reactive oxygen species. Moreover, hHcy due to *MTHFR* gene polymorphism (C677T) was reported as a risk factor of mesenteric venous thrombosis, bowel infarction and has been correlated to colorectal cancer [97]. The increased tHcy level was observed in patients with inflammatory bowel disease. This might be a consequence of the disease itself because sulfur amino acids are metabolized and transported in the gastrointestinal tract. Another study has shown that hHcy causes upregulation of inducible nitric oxide synthase (iNOS; EC 1.14.13.39), which initiates inflammatory changes during hemorrhagic shock resulting in functional and morphological injury of intestine [98]. Hcy affects the activity of matrix metalloproteinases (MMPs), which have an important role in the pathophysiology of several inflammatory disorders of intestine. MMP-2 was found to have the protective function during intestinal inflammation. In contrary, MMP-9 can associated with mucosal damages during inflammatory processes [99]. Therefore, inhibition of MMPs could have a therapeutic potential in targeting intestinal inflammation.

### 4.6. Cancer

For malignant cells, high growth rate is typical and thus higher Met requirement because of increased processes of proteosynthesis and transmethylation. Normal cells can cover their Met consumption from Hcy remethylation. Malignant cells in organs as lung, kidney, breast, colon and bladder are methionine-dependent, because they cannot convert Hcy to Met resulting to Hcy accumulation. An increased level of Hcy is also related to folate concentration. Folate cofactors act as essential intermediates in Hcy remethylation to Met, in SAM synthesis and in the production of nitrogenous bases for DNA/RNA synthesis. In several studies, it was shown that patients with acute lymphoblastic leukemia, colorectal, ovarian, pancreatic, and head and neck squamous cell carcinomas had elevated plasma tHcy level simultaneously [100,101,102]. Methionine-dependent cells have lower SAM/SAH ratio comparing with methionine independent cells. Reduction of intracellular SAM levels can alter cytosine methylation in CpG islands of DNA resulting in the repression of tumor suppressor genes, activation of protooncogenes and also with induction of malignant transformation [1]. Higher level of SAH increased Hcy level as long as Hcy is not converted to Cys by transsulfuration pathways. Several studies observed higher Hcy level and unchanged plasma level of Cys in patients with cancer [101,102]. Naushad et al. [103] analyzed the epigenetic changes that influence cancer progression. They found that hHcy and genetic variants in “one-carbon” pathway have strong influence on the epigenetic profile of two crucial genes, i.e., *RASSF1* and *BRCA1*, thus directly affected breast cancer progression and explaining one of possibilities of the “methionine-dependent phenotype” phenomenon of breast cancer. Inverse association between methylation of *RASSF1* and *BRCA1* loci and lower level of vitamin B12 is translated to clinical setting and it could be a useful public health strategy to decrease the risk of breast cancer [103].

### 4.7. Congenital Disorders

As was mentioned above, high concentration of SAH competes with SAM for the binding site in DNA methyltransferases. This competition is followed by DNA hypomethylation resulting in epigenetic programming [62]. A genome-wide analysis on human fetal cord blood refers to the possible influence of Hcy concentration on the fetal epigenome [104]. Moreover, the effect of hHcy in the developing human fetus has not been fully established yet. It is assumed that impairment of methylation processes during embryogenesis, when the methylation of DNA is reprogrammed, could have a fundamental role in the etiology of malformations in newborns. Furthermore, maternal hHcy, some gene mutations in enzyme of “one-carbon” cycle and low Met levels could also be associated with the increased incidence of congenital disorders. These defects include Down syndrome, congenital heart defect, neural tube defect and nonsyndromic oral clefts [62].

## 5. Conclusions and Perspectives

The imbalance in Hcy metabolism is linked with a number of human pathologies. It remains unclear whether excessive Hcy concentration directly contributes to the pathogenesis of diseases or it represents a biomarker of metabolic aberrations, such as aberrant methyl group metabolism. Different strategies to reduce plasma Hcy concentrations have reached inconsistent results, not just in the case of vascular disorders, but also with respect to neurodegenerative disorders, bone tissue health or cancer. hHcy leads to increased thiolation and homocysteinylation of proteins, both in plasma and in tissues. As a consequence, these post-translational modifications affect the function and activity of different enzymes, like superoxide dismutase, catalase or glutathione peroxidase [34]. Aberrant Hcy metabolism leads to redox imbalance and to increased oxidative stress and formation of reactive oxygen and nitrogen species, followed by the protein, nucleic acid and carbohydrate oxidation, and lipoperoxidation. Moreover, Hcy treatment of cell cultures doubles the rate of telomere shortening. Elevated levels of Hcy can result from the deficiency of one or more enzyme’s vitamin cofactor involved in its metabolism. Therefore, it would be very important to find the strategies to decrease Hcy levels. It was proven that vegan or vegetarian individuals have deficiency in some vitamins, especially B6 and B12 that are involved in remethylation pathway of Hcy. These persons are more sensitive to develop hHcy [105]. Genetic abnormalities and nutritional deficiencies explain only a part of hHcy pathologies. Hormonal and metabolic factors such as diabetes, thyroid diseases and estrogen deficiency interact with Hcy metabolism [106]. In addition, therapy with multiple vitamins and folate might be as a one clue to correct Hcy level in patients. Clinical trials are needed to determine the optimal doses of vitamins.

Neural cells are sensitive to prolonged hHcy treatment, because Hcy cannot be metabolized by transsulfuration pathway or by folate/vitamin B12 independent remethylation pathway. Therefore, Hcy might be used as an additional valuable prognostic and predictive biomarker in neurodegenerative diseases. Elevated level of Hcy detected in cancer patient is one of the major consequences of the rapid tumor cell proliferation. Potentially, circulating Hcy could be used as one of markers to monitor cancer patients during drug therapy, complementing the currently used tumor markers. More studies including larger populations are needed to verify these points and also to find an appropriate cut-off value of Hcy for patients with cancer. Rapid proliferated tumor cells would deplete folate followed by inactivation of “one-carbon” metabolism. Their disability to convert Hcy to Met leads to increased levels of Hcy. This could be one of explanation of “methionine-dependency” of malignant cells. Refsum et al. [107] found elevated Hcy levels in children with acute lymphoblastic leukemia before beginning of drug treatment. It fell dramatically down after few days of cytotoxic drug administration. Potentially, elevated Hcy could be a marker for carcinogenesis and also for detecting recurrence. Sun et al. [102] monitored the Hcy concentrations within the concentrations of the dominant tumor markers in a group of patients with carcinomas, who were not taking drugs. The changes of serum Hcy concentrations parallel the rise and fall with serum tumor markers. These results suggest that serum Hcy, like tumor markers, reflected the tumor cell activity. However, Ozkan et al. [108] observed that higher prevalence of hHcy in lung cancer patients is not sufficient to accept Hcy as a cancer marker. Determination of Hcy in larger cancer population could be important to clarify the usefulness of Hcy as a marker for cancer and its drug therapy.

The epigenetic mechanisms play an important role in elevated Hcy production. Since SAM is a universal donor of methyl group, SAH followed by Hcy are produced during these processes. It becomes more and more evident, that DNA methylation impairment might be suggested as consequence of hHcy caused by endogenous (polymorphisms of genes which code enzymes involved in Hcy and folate pathways) and/or exogenous factors (dietary deficiency of folate and vitamins, protein intake rich in Met and Cys) and may be involved in etiopathogenesis of Hcy toxicity. It is important to find factors that can affect the methyl balance to help us to understand the pathophysiology of diseases from “methylation point of view”. Epigenetic alternations in mitochondrial DNA need to be evaluated in terms of prediction of therapeutic efficacy similar to nuclear DNA [109]. Advanced studies are needed to understand whether and how changes in mitochondrial DNA methylation patterns, global and gene specific are associated to elevated levels of Hcy in context to diseases and risk factors, such oxidative stress, aging and exposure to drugs.

As shown by recent studies, some natural compounds which are able to decrease the plasma level of the Hcy. Resveratrol is a polyphenol compound found in the skin of red grapes, in peanuts and berries. This plant antioxidant can protect the body against damages linked to the increased risk for cardiovascular and neurological diseases or cancer. Resveratrol strongly, but not completely, reduced platelet apoptosis induced by Hcy or Hcy-TL [110]. Another compound, paraoxonase 1 (PON1, EC 3.1.8.1) is a calcium-dependent enzyme synthesized in liver. It is a major component of plasma HDL particles, responsible for the antioxidant protection of HDL or LDL particles. PON1 hydrolyzes Hcy-TL, which is determinant of plasma *N*-homocysteinylated protein concentration [111]. The lactonase (Hcy-thiolactonase) activity of PON1 can help to avoid the post-translational modification of LDL apoproteins through *N*-homocysteinylation in HDL particles. It was confirmed, that another compound, tetrahydrocurcumin which is a herbal antioxidant, improves homocysteinylated cytochrome c mediated autophagy in hHcy mice after cerebral ischemia [112]. Tetrahydrocurcumin may be an effective protective agent in the prevention of oxidative stress induced by hHcy.

In conclusion, the increased prevalence of hHcy in the population and crucial role of elevated Hcy levels in pathogenesis of different diseases make this amino acid an interesting target for future investigations. Advanced studies are needed to understand: (i) the role of new preventative dietary supplements or medicaments, which will decrease plasma Hcy level; (ii) the molecular basis to find the mechanism of Hcy interaction with its target molecules inside the cell also in extracellular space; (iii) the epigenetic alteration of DNA (nuclear and mitochondrial) methylation profiles in correlation with pathogenesis of diseases; and (iv) usefulness and validity of Hcy as a biomarker of multimarker panel to predict (along with other factors such as age, gender, smoking, and some other genetic variants) the risk of developing and/or progression of some diseases.

## Abbreviations

BHMT	betaine-homocysteine *S*-methyltransferase
BBB	blood brain barrier
CBS	cystathionine β-synthase
CKD	chronic kidney disease
CNS	central nervous system
CoA	coenzyme A
CSE	cystathionine γ-lyase
CVD	cardiovascular diseases
Cys	cysteine
DNMT1	DNA methyltransferase 1
GAMT	guanidine-acetate *N*-methyltransferase
ERK	extracellular signal-regulated kinase
GNMT	glycine *N*-methyltransferase
Hcy	homocysteine
Hcy-TL	homocysteine-thiolactone
HDL	high-density lipoprotein
hHcy	hyperhomocysteinemia
LDL	low-density lipoprotein
MAPK	mitogen-activated protein kinase
Met	methionine
MMP	matrix metalloproteinase
MS	methionine synthase
MT	methyltransferase
MTHFR	*N*-5,10-methylene tetrahydrofolate reductase
NADPH	nicotinamide adenine dinucleotide phosphate
NMDA	*N*-methyl-d-aspartate receptor
PEMT	phosphatidylethanolamine *N*-methyltransferase
SAH	*S*-adenosyl-l-homocysteine
SAM	*S*-adenosyl-l-methionine
Ser	serine
tHcy	total homocysteine
TIMP	tissue inhibitor of metalloproteinase
THF	tetrahydrofolate
